# EMBRACING CONNECTIVITY: TANGIBLE, CUSTOMIZED CHECK-INS FOR OLDER ADULTS TO COMBAT SOCIAL ISOLATION

**DOI:** 10.1093/geroni/igae098.1653

**Published:** 2024-12-31

**Authors:** Pallabi Bhowmick, Lesa Huber

**Affiliations:** University of Illinois Urbana-Champaign, Mahomet, Illinois, United States; Indiana University Bloomington, Bloomington, Indiana, United States

## Abstract

Increasing social isolation among older adults poses significant concerns for health and well-being. While older adults consider families as their strongest bonds, friends and neighbors often play a pivotal role as the primary support network. This study investigates the efficacy of leveraging peer support through a tangible, customized daily check-in to prolong independent living. The study engaged 15 older adults aged 70-90 in a 3-hour, recorded co-design session with the objective of ensuring their needs were considered early in the design process. Transcripts were coded and thematically analyzed to reveal three key findings. Firstly, 80% of participants lived alone and strongly acknowledged the need for a daily check-in. They noted, neighbors and peers in close proximity often serve as the first line of support. Secondly, 87% emphasized the importance of a customized interface aligned with their personal routine, (e.g. checking-in by making tea), affirming it would enhance usability. Additionally, tangible user interfaces integrate digital information into everyday objects, providing familiar, intuitive user interfaces. Participants concurred this could address sensory-motor challenges and mitigate the learning curve associated with digital interfaces. Thirdly, all participants strongly opposed one-way passive monitoring, advocating for a peer-to-peer check-in system that allows older adults to take care of each other instead of always being on the receiving end of care. This promotes reciprocity, inclusivity, and reduces stigma. Tangible, customized, peer-based check-in systems have the potential to improve social connection, address ageism in technology design, and encourage technology acceptance and adoption among the older adult population.

